# When Elderly Outperform Young Adults—Integration in Vision Revealed by the Visual Mismatch Negativity

**DOI:** 10.3389/fnagi.2017.00015

**Published:** 2017-01-31

**Authors:** Zsófia Anna Gaál, Flóra Bodnár, István Czigler

**Affiliations:** ^1^Institute of Cognitive Neuroscience and Psychology, Research Centre for Natural Sciences, Hungarian Academy of SciencesBudapest, Hungary; ^2^Doctoral School of Psychology, Eötvös Loránd UniversityBudapest, Hungary; ^3^Institue of Psychology, Eötvös Loránd UniversityBudapest, Hungary

**Keywords:** aging, visual mismatch negativity, inhibition, visual persistence, ERP

## Abstract

We studied the possibility of age-related differences of visual integration at an automatic and at a task-related level. Data of 15 young (21.9 ± 1.8 years) and 15 older (66.6 ± 3.5 years) women were analyzed in our experiment. Automatic processing was investigated in a passive oddball paradigm, and the visual mismatch negativity (vMMN) of event-related brain potentials was measured. Letters and pseudo-letters were presented either as single characters, or the characters were presented successively in two fragments. In case of simultaneous presentation of the two fragments (whole character) vMMN emerged in both age groups. However, in successive presentation vMMN was elicited only by the deviant pseudo-letters, and only in the older group. The longest stimulus onset asynchrony (SOA) in this group was 50 ms, indicating longer information persistence in elderly. In a psychophysical experiment, the task was to indicate, which member of a character pair was a legal letter. Again, the letters and pseudo-letters were presented as fragments. We obtained successful integration at 30 ms (0 ms interstimulus interval), but not at longer SOAs in both age groups, showing that in case of task-relevant stimulation level there was no detectable age-related performance difference. We interpreted the results as the efficiency of local inhibitory circuits is compromised in elderly, leading to longer stimulus persistence, and hence better visual perception in this particular case.

## Introduction

Age-related changes of visual perception are a field of considerable research activity at the levels of sense organs and cognitive functions (for reviews see Loftus et al., 1987; Owsley, 2011). Fewer studies have investigated the changes at the level of elementary information processing, such as the possibility of compromised temporal acuity of perceptual processes in vision. The available studies on visual persistence concentrated on three fields: temporal integration (Kline and Baffa, 1976; Kline and Orme-Rogers, 1978; Di Lollo et al., 1982), visual masking (Walsh, 1976; Cramer et al., 1982; Di Lollo et al., 1982; Roinishvili et al., 2011), and fusion frequency (McFarland et al., 1958). These studies applied methods of psychophysics. Psychophysical measurements require attentional processing of the target stimuli, therefore such studies cannot isolate the contribution of automatic (sometimes labeled as pre-attentive) processes from the attentional effects. However, at least in the auditory modality, task-related processing on temporal acuity had a marked influence on age-related differences (Bertoli et al., 2002). In psychophysical measures gap detection threshold was similar in the two age-groups, but automatic processing measured by the mismatch negativity (MMN) component of event-related potentials (ERP) indicated a considerable age-related difference. The aim of the present study was to investigate age-related differences in visual temporal acuity at the level of automatic processing. We introduced a visual integration task, and recorded an indicator of automatic change detection, the visual mismatch negativity (vMMN) component of ERP of brain electric activity.

A behavioral measure of the longer persistence in elderly is the integration method. An interesting feature of the integration method is that longer persistence (decreased visual acuity) results in better performance. In integration studies parts of the stimuli are presented successively, and in order to recognize the stimuli, temporal integration of the two halves is necessary. The longer the critical temporal gap is between the parts for achieving successful recognition or discrimination, the longer the persistence, or in other words, the worse the temporal acuity is. In the first experiment in the field Kline and Baffa (1976) did not find longer persistence in the elderly. Kline and Orme-Rogers (1978) attributed the failure to the stimuli of the task. They argued that the to-be-integrated word fragments were constructed from dot patterns, and the older group had specific difficulty to integrate the dots into letters. Therefore, Kline and Orme-Rogers (1978) presented letter fragments of three-letter words as straight horizontal and vertical lines. In this experiment the longest integration time of the older group was longer than the duration in the younger group. Di Lollo et al. (1982) obtained convergent results in a task with straight vs. broken line segments, and in discrimination task with full vs. incomplete dot matrices However, contrary to these findings, in a study by Walsh and Thompson (1978) elderly participants reported a shorter threshold duration for reporting a continuous stimulus vs. two successive ones. Di Lollo et al. (1982) attributed the difference to age-related response bias factors. In summary, despite opposite results, integration studies indicated longer persistence in the elderly, and the longer persistence could result in better performance in some studies.

Speculating about the underlying mechanisms of increasing integration duration in the older group, there are two possible explanations. The first one is the longer passive activity decay of the neural network responsible for the persistence; the second one is the less efficient process responsible for the active termination of persistence in older age. The second possibility is more attractive, because passive decay is not supported by a justifiable neurophysiological mechanism. The possibility of active mechanism is related to the general notion on cognitive aging. Age-related cognitive impairments are frequently explained with deficits of inhibitory processes (Hasher et al., 1991), but this theory does not differentiate between the various types of inhibition. However, compromise of a lower level inhibitory process can be the reason for longer integration duration, and this explanation is related to well-established brain mechanisms. Throughout the brain the primary cells communicate with interneurons. Interneurons have both feedback and feedforward inhibitory influence on the primary neurons (for review see Kullmann et al., 2012; Fino et al., 2013). This way there is a temporal limit on the activity of any circuits. Age-related decrease of the efficiency of the inhibitory neurotransmitter (GABA) is well documented in the visual system (for a review see Lehmann et al., 2012), but the locus of inhibitory influence of various types of interneurons is different (Miyamoto et al., 2012), and there are species-specific differences.

The present study was based on the integration study by Kline and Orme-Rogers (1978), and applied their method into the vMMN paradigm. vMMN is elicited by infrequent (deviant) stimuli appearing within sequences of frequent (standard) ones, as a difference potential between the ERPs elicited by the standard and deviant. The difference is due to the decreased activity to the repeated standard (stimulus-specific adaptation; e.g., May and Tiitinen, 2010; O’Shea, 2015), and an increased activity to the deviant (genuine mismatch; e.g., Schröger and Wolff, 1996; Czigler et al., 2002; Kimura et al., 2009). Genuine vMMN is elicited by non-attended (irrelevant) stimuli, and considered to be an index of automatic detection of violated sequential regularities. vMMN is elicited by deviant visual features (color, spatial frequency, motion direction, orientation, etc.), object-related characteristics, facial emotions, and categorical differences (for reviews see Czigler, 2007; Kimura, 2012; Stefanics et al., 2014). One of the category-related effects is especially important in the present context. Sulykos et al. (2015) presented legal letters and pseudo-letters as standards and deviants (and vice versa). The letters and pseudo-letters as deviant characters elicited vMMN, but the effect was asymmetric, vMMN latency to illegal characters had shorter latency. In the present study we capitalized on these results. We presented letters and pseudo-letters, but in the temporal integration sequences the letters were cut into two fragments. The main variable was the stimulus-onset asynchrony (SOA) between the fragments. We assessed the longest SOA when vMMN emerged for the deviant category (either letter or pseudo-letter) of the sequence, and compared this SOA across the participants’ ages.

We know only five studies in which vMMN was compared between older and young participants, but the results are equivocal (for a review see Kremláček et al., 2016). For motion direction deviancy Lorenzo-López et al. (2004) obtained decreased vMMN amplitude in older participants, and in this group vMMN appeared over a smaller set of scalp locations. Tales et al. (2002) presented task-irrelevant single and double bars as standard and deviant stimuli, and obtained smaller vMMN in the older participants. In a similar paradigm Tales and Butler (2006) compared healthy old participants and Alzheimer’s disease (AD) patients. The vMMN amplitude of the healthy older group was smaller than the amplitude of the younger group. Interestingly, in a later phase of the session vMMN amplitude of the AD group increased. However, in the third study of this group with the same paradigm, Stothart et al. (2013) did not find age-related differences. Finally, Iijima et al. (1996) did not report age-related differences in an oddball task with shape discrimination. Apart from the Lorenzo-López et al. (2004) study, the control for the involvement attentional effects was not particularly strict. In the Tales et al. (2002), Tales and Butler (2006), and Stothart et al. (2013) studies the task-relevant and vMMN-related shapes were simultaneously presented as a single stimulus, and in the Iijima et al. (1996) study there was no attempt for control of attention. To obtain further data on the possibility of age-related vMMN changes, we added a condition with unitary presentation of the letters and pseudo-letters.

Finally, to compare the automatic processes of visual integration to the task-related time window of integration, we conducted an experiment using the fragmented characters in a psychophysical measurement.

We proposed that at 0 ms SOA (traditional passive oddball paradigm) the vMMN emerges in both groups. We hypothesized that in older adults the decreased inhibitory control results in longer persistence compared to young adults. Consequently, the older group can detect the difference between standard and deviant stimuli at longer SOAs than the young group, indicated by the emerging vMMN. Longer persistence also leads to better performance in the psychophysical experiment at longer SOA values in older compared to young adults.

## Materials and Methods

### Participants

Participants were drawn from a larger pool (30 older and 29 younger adults) with the criterion of a reliable N1 component at Oz location. This selection was independent of the vMMN related data. Due to technical failures the results of two older and three younger participants were omitted. This way there were 15 participants in each group. Full scale Wechsler IQ was conducted in both groups in a separate session (young adults: 21.9 ± 1.8 years, IQ = 100.0 ± 20.0, older adults: 66.6 ± 3.5, IQ = 115.0 ± 18.7). Participants had normal or corrected-to-normal vision measured by the Kettesy type decimal visual acuity table (a Hungarian variant of the Snellen chart), and according to their statement they had no history of any kind of neurological or psychiatric disease.

The Joint Psychological Research Ethics Committee (Hungary, 42/2015) approved the protocol of the experiment, the study was carried out in accordance with the recommendations of the Declaration of Helsinki and a written informed consent was obtained from all participants.

### Electrophysiological Study

#### ERP-Related Stimuli and Stimulus Sequences

**Figure 1:**
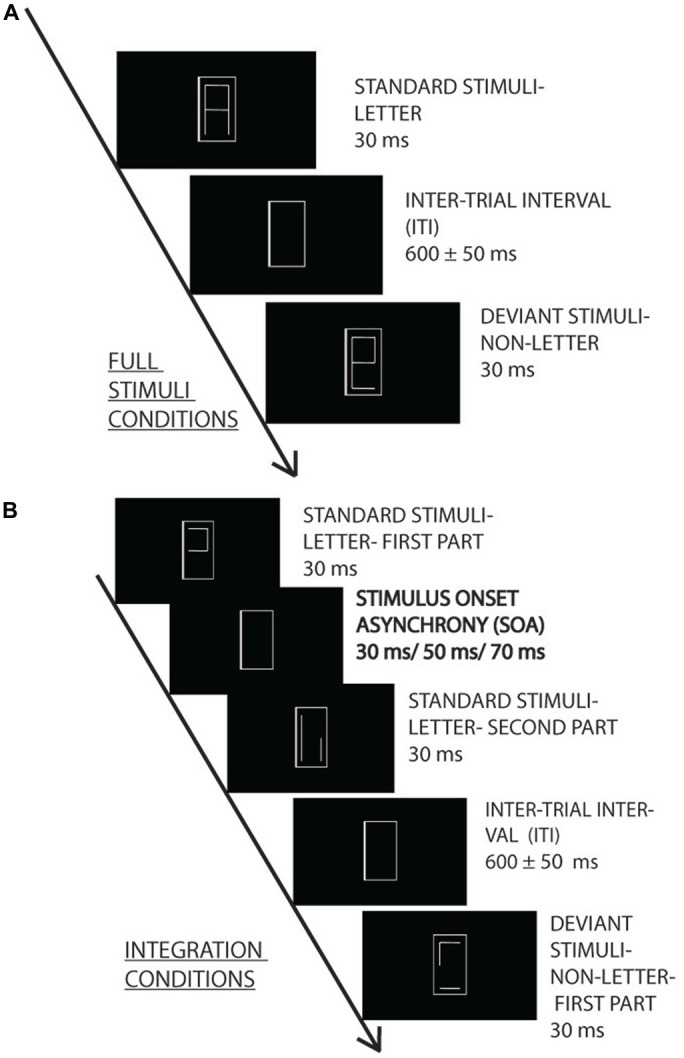
**Experimental design.** Irrelevant letter and pseudo-letter stimuli appeared within a gray frame with 600 ± 50 ms intertrial interval (ITI). The task was to press a button when the frame became thicker. The irrelevant standard (80%) and deviant (20%) characters were presented as a whole **(A)** or in two fragments **(B)** with 30, 50 and 70 ms stimulus onset asynchrony (SOA) (0, 20 and 40 ms interstimulus interval).

The stimuli were two letters and two pseudo-letters, irrelevant for the ongoing task. Letters and pseudo-letters elicited vMMN in studies by Sulykos et al. (2015) and Amado and Kovács (2016). Each character was segmented into two parts in five different ways. Figure 1 shows the characters and examples of the segments. The trials within stimulus sequence consisted of pairs of two fragments. The letters or pseudo-letters appeared at the center of a 19″ CRT monitor (LG 915FT Plus). The characters had seven segments, and letters as whole appeared within an area of 0.78° × 1.75°; the line thickness was 0.07°, from a 150 cm viewing distance. The stimuli were presented with white (90.1 cd/m^2^), and the background was black (0.1 cd/m^2^).

The letter fragments were presented for 30 ms. In the various SOA conditions the SOAs were 0, 30, 50 and 70 ms. The interval between the stimulus pairs was 600 ms ± 50 ms random jitter.

Oddball sequences were created from the characters, with either the letters or the pseudo-letters as the infrequent (deviant) stimuli. The probability of the deviants was 0.2. Within a stimulus sequence there were 250 stimuli. The two versions of the standard and deviant stimuli had equal probability. Accordingly, each standard had the probability of 0.4, and each deviant had the probability of 0.1. There were four sequences at each SOA level, and in half of the sequences the letter, in the other half of the sequences the pseudo-letter was the deviant. Accordingly, 16 sequences were presented (four SOAs, two types of deviancy, repeated twice). The order of sequences was random.

#### Task and Task-Related Stimuli

The characters appeared within a gray frame (1.26° × 2.51°) with a 0.057° line thickness; from a viewing distance of 150 cm (frame luminance: 37.56 cd/m^2^). The choice of central presentation of the characters surrounded by the task-related stimuli followed the method of Urakawa et al. (2010), and the aim of this choice was to improve the sensibility to the irrelevant stimulus change. From time to time one of the sides of the frame became thicker (0.1°), and participants had to indicate this change. The duration of thickening was 300 ms, and the frequency of the change was 10 s (between 5 and 15 s). This way approximately 20 changes occurred in a block. The changes were balanced between the sides of the frame, and the change never occurred during the presentation of the deviant stimuli, or during the presentation of the last standard before the deviant. During the task, reaction time and hit rate were measured to each frame changes.

#### Measurement of Brain Electric Activity

Brain electric activity was recorded (bandpass filter: DC-70 Hz; sampling rate: 1000 Hz; SynampsRT amplifier, NeuroScan recording system) with Ag/AgCl electrodes placed at 37 locations according to the extended 10–20 system by using an elastic electrode cap (EasyCap). The reference electrode was on the nose tip, the ground was at FCz. Horizontal EOG was recorded with a bipolar configuration between electrodes positioned lateral to the outer canthi of the eyes. Vertical eye movement was monitored with a bipolar montage between electrodes placed above and below the left eye. The EEG signal was bandpass-filtered offline, with cutoff frequencies of 0.1 Hz and 30 Hz (24 dB slope). Segmentation was performed from −100 ms to +600 ms relative to the onset of the first fragment. It was followed by a baseline correction (pre-stimulus interval). Note that in the statistical analysis time point 0 ms is defined as the onset of the second fragment. Epochs with an amplitude change exceeding ±100 μV on any channel were excluded from further analysis. ERPs were averaged separately for the standard and deviant stimuli in each condition. To identify change-related activities, ERPs elicited by standard stimuli were subtracted from ERPs elicited by deviant stimuli in the opposite condition (Kujala et al., 2007).

#### Data Analysis

vMMN is expected to emerge as a posterior negativity in the 150–300 ms range after the onset of deviancy, i.e., after the onset of the second fragment. To identify vMMN we constructed two regions of interest (ROIs) from PO8 and PO10 (right side) and PO7 and PO9 (left side), respectively. These ROIs were chosen on the basis of the usual posterior distribution of vMMN. The criteria for vMMN appearance was at least 20 subsequent significant *t*-values (20 ms) in the difference potentials (against zero, *p* < 0.05) within the expected range (150–300 ms), at least in one of the ROIs. Latencies and amplitudes of the vMMNs between the two groups were compared in the 0 ms SOA condition in repeated measures of ANOVAs (Statistica 12 software). vMMN latencies were measured as the latency of the largest negative values within the 150–300 ms range, and the amplitudes were measured as the mean values of a 20 ms epoch around the largest negativity of the group average. Amplitudes and latencies were compared in ANOVAs only in cases where the *t*-values indicated vMMN emergence at least in one of the ROIs. Note that 0 ms condition was a typical oddball sequence. In this condition we compared the vMMN in the two age-groups. The factors were *Age* (older and younger) and *ROI* (left and right). To evaluate temporal changes, the *SOA* (0, 30, 50 ms) factor was involved into the ANOVA. To assess the performance of the thickness detection task we calculated hit rates. These values were compared in ANOVAs with factors of *Age* and *SOA* (0, 30, 50, 70 ms). In *post hoc* tests we applied the Tukey HSD procedure. When appropriate we used the Greenhouse-Geisser correction. Effect sizes are indicated by partial eta squared ($\eta_{\text{p}}^{2}$).

### Psychophysical Experiment

After the EEG session a psychophysical task was introduced. Participants decided which member of a stimulus pair (same stimuli as in the electrophysiological task) was a letter or a pseudo-letter. Like in the EEG session, both members of the pairs consisted of two fragments. Duration of the fragments were 30 ms, and the SOA (onset-to-onset interval between the fragments) was 30, 50, 70 ms, respectively. In a two-alternative forced choice paradigm participants decided which member of the character pair was the letter. They pressed a button according to their choice after which the next trial was presented. The experiment contained 164 trials, the trials were balanced according to SOA (30, 50, 70), and the order of the letter and pseudo letter within the pair. Results of the two-alternative forced choice task were calculated as the number of correct choices, and analyzed in an ANOVA with factors of *Age* and *SOA*.

## Results

### Event-Related Potentials

#### Exogenous Components

**Figure 2:**
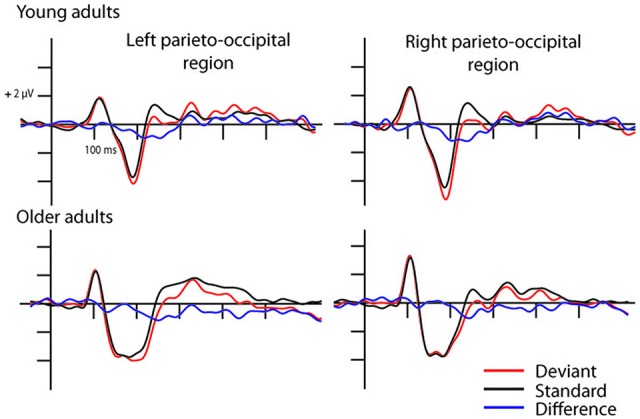
**Event-related potentials (ERPs) in the full stimuli pseudo-letter condition.** Visual mismatch negativity (vMMN) emerged both in young (upper row) and older adults (lower row) as the deviant minus standard ERP difference wave shows in the 150–300 ms time window.

In the full stimulus condition two exogenous components (P1 and N1) emerged. Figure 2 shows the ERP in the two age groups for the deviant and standard stimuli, and Table 1 shows the amplitude and latency values of these components. According to ANOVAs with factors of *Age*, *Stimulus* (deviant, standard), *Character* (letter, pseudo-letter) and *Side* (left, right) P1 amplitude was generally larger at the right side (*F*_(1,28)_ = 14.67, *p* < 0.001, $\eta_{\text{p}}^{2}$ = 0.34). In the N1 range; the activity of the two groups was different, the group average N1 was wider in the older group. We obtained no main effect of age group, but the N1 latency was shorter for the letters compared to pseudo-letters (*F*_(1,28)_ = 6.45, *p* < 0.05, $\eta_{\text{p}}^{2}$ = 0.19). This effect of character was seen in the older but not the younger group (*F*_(1,28)_ = 5.00, *p* < 0.05, $\eta_{\text{p}}^{2}$ = 0.15). In the context of the present study, it is more important that in spite of the apparent differences, deviancy effects in the full stimuli condition were fairly similar.

**Table 1:** **Amplitude and latency data of P1 and N1 components (mean and standard deviation) in the various conditions in young and older adults**.

		P1		N1	
Condition	ROI	Amplitude (μV)	Latency (ms)	Amplitude (μV)	Latency (ms)
**Young adults**
Letter, Deviant	Right	2.28 ± 1.71	105.4 ± 13.6	−3.93 ± 2.51	185.5 ± 9.5
	Left	1.59 ± 1.85	106.5 ± 19.3	−3.37 ± 1.71	187.3 ± 12.2
Letter, Standard	Right	2.25 ± 1.68	105.1 ± 10.1	−3.85 ± 2.96	182.5 ± 14.6
	Left	1.12 ± 1.34	111.4 ± 14.4	−3.54 ± 2.86	185.0 ± 16.3
Pseudo-letter, Deviant	Right	2.42 ± 1.60	106.9 ± 8.7	−5.23 ± 3.80	187.2 ± 10.1
	Left	1.73 ± 1.65	112.0 ± 8.7	−4.09 ± 3.01	185.5 ± 16.2
Pseudo-letter, Standard	Right	2.54 ± 1.42	105.5 ± 8.8	−4.40 ± 3.27	182.3 ± 13.1
	Left	1.67 ± 1.32	112.9 ± 10.9	−3.59 ± 2.76	187.2 ± 12.8
**Older adults**
Letter, Deviant	Right	3.32 ± 2.16	110.8 ± 9.0	−4.04 ± 2.32	173.0 ± 17.4
	Left	2.51 ± 1.55	109.8 ± 10.1	−3.54 ± 2.90	175.3 ± 16.2
Letter, Standard	Right	3.08 ± 2.37	110.2 ± 12.1	−3.42 ± 2.24	177.0 ± 16.2
	Left	2.39 ± 1.60	110.9 ± 9.3	−3.35 ± 2.69	179.2 ± 17.7
Pseudo-letter, Deviant	Right	3.21 ± 1.97	109.8 ± 9.7	−3.52 ± 2.74	191.5 ± 25.5
	Left	2.25 ± 1.57	101.5 ± 8.4	−3.99 ± 2.54	193.5 ± 18.3
Pseudo-letter, Standard	Right	3.07 ± 1.98	109.5 ± 8.4	−3.74 ± 2.37	173.8 ± 17.3
	Left	2.15 ± 1.26	109.3 ± 8.8	−3.74 ± 2.68	176.2 ± 18.2

#### Difference Potentials

In case of the 0 ms conditions vMMN emerged to pseudo-letter stimuli in both groups, and to letter stimuli in case of the older adults. In the 30 and 50 ms conditions vMMN was elicited only in the older group^1^. In the 70 ms condition, no vMMN emerged in the two groups (Table 2 contains the significant temporal windows in the two ROIs).

**Table 2:** **Visual mismatch negativity (vMMN) results**.

SOA (ms)	Age group	Stimulus	ROI	Temporal window (ms)
0	Older	Letter	Right	150–180
0	Young	Pseudo-letter	Right	190–248
0	Older	Pseudo-letter	Right	227–255
			Left	217–270
30	Older	Pseudo-letter	Right	243–268
			Left	212–253
50	Older	Pseudo-letter	Right	211–252
			Left	219–256

*The time range of significant t-values in various conditions, age groups and ROIs for letter and pseudo-letter stimuli*.

##### Whole characters (0 ms SOA)

**Figure 3:**
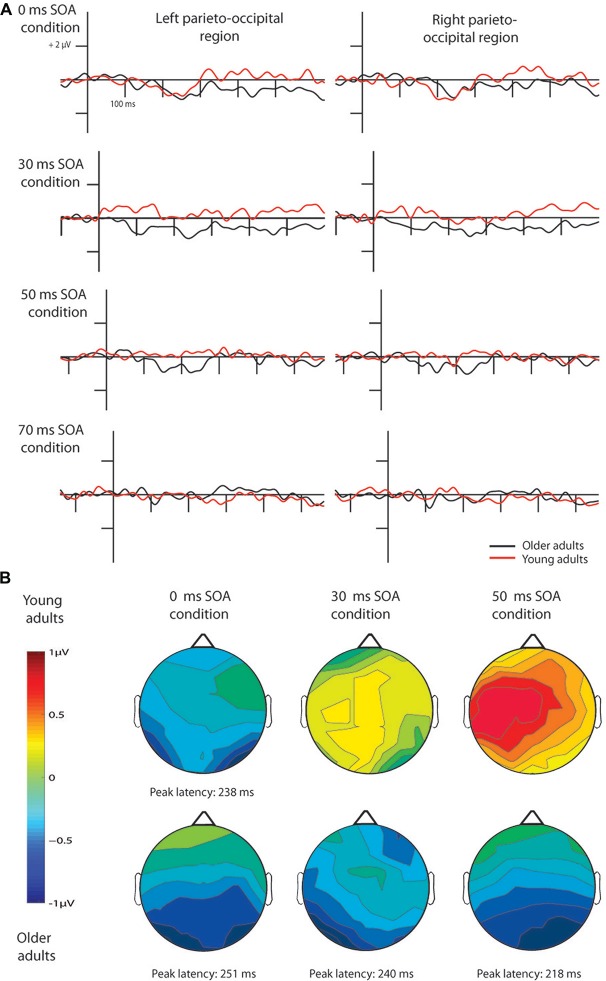
**Difference waves for pseudo-letters in the four conditions (A)** and their scalp distribution **(B)**. vMMN emerged in young adults only in the 0 ms SOA condition, while in older adults in the 0, 30 and 50 ms conditions. Note that ERPs are shown 100 ms before the 1st stimulus in all cases, but time point 0 ms is at the beginning of the 2nd fragment, hence the y axes are at different positions according to the different SOAs.

The deviant minus standard difference potentials appeared as a posterior negativity in the older group for both deviant pseudo-letters and letters, and in the younger group only for deviant pseudo-letters. Figure 3 shows the difference potentials and their scalp distribution in the two age groups. *T*-tests indicated significant differences (20 subsequent significant values) in the older group at 0 ms SOA for letters (right ROI) and pseudo-letters (right and left ROIs), in the younger group for pseudo-letters, right ROI, at 30 ms SOA in the older group for pseudo-letters (right and left ROIs), at 50 ms SOA in the older group for pseudo-letters (right and left ROIs). Furthermore, the negativity had apparently longer duration in the older group in the left ROI than in the right ROI, and longer than in the younger group. However, there were no significant 20 ms periods above the latency value of 300 ms in any conditions. Table 3 shows the latency values of the largest negativity and amplitude values (mean values of the 20 ms periods around the peak). To analyze the effects of *Age* and *Side* differences, we compared the latencies of the maximal negativity, and the amplitude values around the amplitude maxima (10 ms ranges around the maxima) in ANOVAs with *Age* and *ROI*. vMMN peaked earlier in the younger group (*Age* main effect, *F*_(1,28)_ = 5.56, *p* < 0.05, $\eta_{\text{p}}^{2}$ = 0.17). There were no other significant effects. As for the amplitudes, *Age × Side* interaction was significant (*F*_(1,28)_ = 4.81, *p* < 0.05, $\eta_{\text{p}}^{2}$ = 0.15), but *post hoc* analysis did not reveal pairwise differences.

**Table 3:** **Amplitude and latency data of the vMMN (mean and standard deviation) in those conditions in which the deviancy was significant**.

		vMMN	
Condition	ROI	Amplitude (μV)	Latency (ms)
**Young adults**
0 ms	Right	−1.20 ± 1.86	234.2 ± 24.3
	Left	−0.83 ± 1.80	235.4 ± 19.9
**Older adults**
0 ms	Right	−0.98 ± 1.44	249.6 ± 16.9
	Left	−1.16 ± 1.45	253.3 ± 15.6
30 ms	Right	−0.75 ± 1.64	245.7 ± 19.9
	Left	−1.20 ± 1.85	240.5 ± 20.7
50 ms	Right	−1.03 ± 1.02	226.5 ± 19.3
	Left	−0.92 ± 1.50	223.9 ± 17.7

##### Stimulus onset asynchrony (SOA) effects

In the younger group, we obtained no deviant-related difference at 30, 50 and 70 ms SOAs. Accordingly, the *t*-tests indicated no differences. On the contrary, in the older group there were negativities in the 30 and 50 ms conditions. At 70 ms SOA no difference emerged (see Figure 3). Table 2 shows the ranges of significant differences (at least 20 ms ranges).

In the older group, we compared the amplitudes and latencies between the two sides and different SOAs. For the amplitude we did not found significant main effect or interaction. The latency was shorter in the 50 ms SOA condition compared to 0 and 30 ms conditions (*SOA* main effect: *F*_(2,28)_ = 6.66, *p* < 0.01, $\eta_{\text{p}}^{2}$ = 0.32).

### Behavioral Results

The task during EEG registration was to indicate the thickening of one of the frame sides We found a significant *SOA* × *Age* interaction (*F*_(3,84)_ = 2.84, *p* < 0.05, $\eta_{\text{p}}^{2}$ = 0.09), but the Tukey HSD *post hoc* test showed significant differences only between 0 and 50 ms SOA conditions (*p* < 0.05) in the young group. Hit rates of the two age groups in the various conditions are presented in Table 4.

**Table 4:** **Performance of young and older adults in the EEG task (hit rate in percent—mean with standard deviation)**.

	0 ms SOA	30 ms SOA	50 ms SOA	70 ms SOA
**Young adults**	76.6 ± 7.1	82.4 ± 6.5	84.9 ± 6.1	83.4 ± 8.6
**Older adults**	78.2 ± 12.7	77.1 ± 14.8	77.1 ± 14.5	80.1 ± 11.0

### Psychophysical Experiment

**Figure 4:**
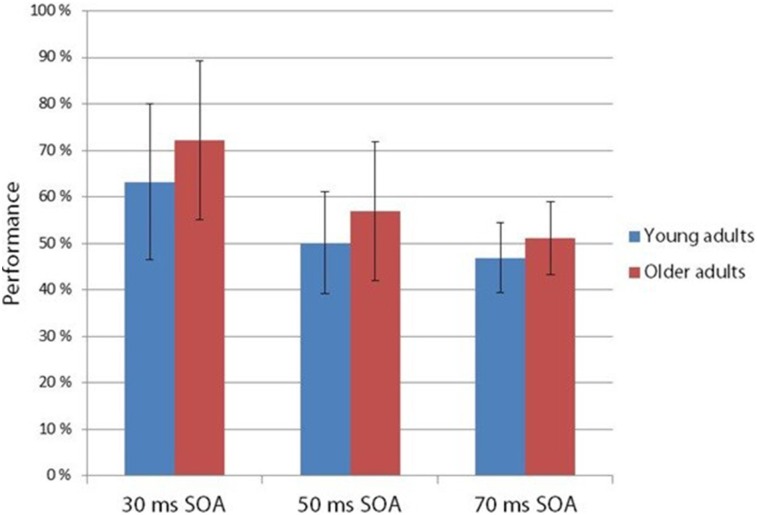
**Hit rate in the two-alternative forced choice task at 30, 50 and 70 ms SOA in young and older adults.** Age groups did not differ in their performance, but SOA main effect was found: performance deteriorated in the 30, 50 and 70 ms SOA order (Vertical bars denote standard deviations).

The two-alternative forced choice experiment measured the behavioral level of performance (see Figure 4). According to a two-way ANOVA with factors of *Age* and *SOA*, the main effect of *SOA* was significant (*F*_(2,54)_ = 35.73 *p* < 0.001, $\eta_{\text{p}}^{2}$ = 0.57). No other effects were significant. At 30 ms SOA the integration was fairly successful, but declined to chance level at 50 ms.

## Discussion

The aim of the present experiment was the investigation of deviant-related effects (vMMN), and thus the analysis of the possibility of age-related temporal integration changes. It is important to emphasize that vMMN in the present paradigm is an index of an automatic process assuming the categorical processing (letter vs. pseudo-letter) of task-irrelevant stimuli. vMMN emerged in the 0 ms SOA condition (i.e., in the condition where the whole characters appeared within a single stimulus). In the older group vMMN was present for the deviant letters and pseudo-letters, but in the younger group only to the deviant pseudo-letters. The results of the younger group are closely related to our previous finding on perceptual categorization (Kecskés-Kovács et al., 2013), where random patterns among symmetric ones elicited vMMN, but not vice versa. We argued that symmetry acquired a category, and the appearance of random patterns violated the regulation of the category. On the contrary, “randomness” is non-categorical; therefore a symmetric pattern cannot violate a category. The same way of reasoning can be applied to the present results of the younger group, in which unlike the pseudo-letters, familiar characters were treated as a category, and the appearance of non-familiar characters violated the sequential appearance of categorized stimuli. It seems that in the older group the process was different. There were two frequent and two infrequent stimuli, and the infrequent ones, independent of familiarity, elicited vMMN. vMMN to two standards and two deviants is not unprecedented in the vMMN research (Winkler et al., 2005).

In the full stimulus condition the peak amplitude was similar in the older and younger participants, but the latency of the larger negativity was longer in the elderly. In this respect our results were similar to some auditory MMN findings (Bertoli et al., 2002; Correa-Jaraba et al., 2016). In the older group deviant minus standard difference emerged as a long-lasting negativity, however, the difference was significant only between 200 ms and 300 ms. The long-lasting negativity for deviant minus standard ERPs is not unique in vMMN studies (Tales et al., 2002; Lorenzo-López et al., 2004; Zhao and Li, 2006); Sel et al., 2016; Urakawa et al., 2010). In the younger group the differences restricted to the 200–300 ms time window. When the to-be-integrated stimuli were task-related, Kline and Orme-Rogers (1978), Walsh and Thompson (1978) and Di Lollo et al. (1982) obtained longer stimulus persistence in older participants. This is exactly the result we obtained in our vMMN paradigm. In the younger group vMMN emerged only at 0 ms SOA, whereas in the older group we found vMMN at 0, 30 and 50 ms SOA values. Accordingly, even at the level of the automatic detection of violation of a sequential regularity, integration time was longer in the older participants. It is important to note that this result cannot be explained as a pure low-level phenomenon of the visual system, because at 30 and 50 ms SOA values the effect was asymmetric even in the older group, vMMN emerged only for pseudo-letters. It means that stimulus familiarity, a long-term memory influence contributed to the obtained change detection effect. Acquired language-related categorization influences vMMN to elementary visual dimensions like color (Thierry et al., 2009; Athanasopoulos et al., 2010). However, as an alternative possibility, in the present study the locus of the familiarity was at a lower level, on the level of object/gestalt formation. As some results show, vMMN is sensitive to object-related characteristics (Müller et al., 2010, 2013). Familiarity may facilitate gestalt formation (Sassi et al., 2014), therefore letters would have advantage over the pseudo-letters. On the basis of gestalt formation, vMMN only for pseudo-letters in the younger group and the longer integration period for the pseudo-letters in the older group seem to be a tenable alternative to the semantic effect. This explanation corresponds to the results Kline and Baffa (1976), showing that older participants have difficulty in integrating simultaneously presented stimulus elements, but stimulus familiarity improves the stimulus integration.

However, unlike in the Kline and Orme-Rogers (1978), Walsh and Thompson (1978) and Di Lollo et al. (1982) studies, in our behavioral two-alternative forced choice task we did not find longer persistence in the older group. When presenting the fragments one after the other without an empty period (30 ms SOA), detection performance was well above chance in both age groups, but performance sharply decreased at 50 ms SOA. Di Lollo et al. (1982) investigated the appearance or absence of a gap within lines and detection of a missing element in a dot matrix. Walsh and Thompson (1978) investigated the integration experience of two circles (one or two circles in succession), and in the Kline and Orme-Rogers (1978) study the task was the identification of familiarized words. The task of the present study was more similar to that of the Di Lollo et al. (1982) study, whereas the stimuli were more similar to the stimuli of the Kline and Orme-Rogers (1978) study. The combination of the different stimuli task demands may lead to different results. At any rate, in our behavioral task the younger group did not outperform the older participants.

Unfortunately, our knowledge about the brain mechanism responsible for the automatic integration processes is limited. The asymmetry of vMMN emergence between the letters and pseudo-letters (i.e., the difference between a familiar and unfamiliar stimulus category) shows that this activity is beyond the lowest levels of visual processing. The broad parieto-occipital surface distribution of the vMMN indicates the involvement of a larger neural network.

Decreased efficiency of inhibitory function is a viable explanation of cognitive changes (e.g., Hasher et al., 1991). In the introduction we suggested that the longer integration duration can be a consequence of less active GABAergic activity of inhibitory circuits. However, to prevent unsubstantiated generalization, it is important to note that the possibility of compromised inhibitory activity as an underlying deficit in temporal acuity in elderly is different from the inhibitory processes in the fields of working memory, attention and response withdrawal. In case of elementary visual processes inhibition may be realized by local modality specific circuits, in other fields of cognitive activity inhibition is due to the activity of distant (mainly anterior) neural structures (Turner and Spreng, 2012). Overall, we have the tentative conclusion that inhibitory processes deteriorate in older adults at the early stages of visual processing too.

As for the longer integration time of the older participants at vMMN level, and the similar performance in psychophysics, it is important to note that the role of attention was different in the two paradigm. Attention may amplify the efficiency of perceptive and post-perceptive processes, but also may generate reappraisal circles. The former effect of attention may increase performance in the younger group, whereas reappraisal may add unwanted noise to the perceptual processes, and this way decrease performance in the older group. Inevitably, this *post hoc* explanation generates testable possibilities on age-related perceptual changes.

Analysis of the exogenous activity was beyond the primary purpose of this study. This is because scalp-recorded activity is a summary of various sources (Di Russo et al., 2002), and the sub-components are differentially sensitive to stimulus features of the various visual features across the studies, and also for the attention demands of the particular tasks. As for the results on stimuli unrelated to attentional demand, in some studies larger P1 emerged in the older group (Zalar et al., 2015 to motion onset stimuli, and Daffner et al., 2013 to letter onset), but highly different results appeared for color square stimuli (Čeponiené et al., 2008). In the present study larger positivity emerged at the right side. The age-related amplitude difference was not reliable. Concerning N1, De Sanctis et al. (2008) obtained broader N1 in an older group, and in this respect their result was similar to the present one. In previous studies various age-related N1 amplitude differences emerged (for a summary Daffner et al., 2013; Zalar et al., 2015). Therefore, we conclude that the equivocal findings on the field of exogenous visual components do not provide a proper data base for theorizing on age-related processing differences.

## Conclusion

We obtained category-related (letters vs. pseudo-letters) vMMN in older and younger adults, separated by a temporal gap. If stimuli were presented in two fragments, only pseudo-letters in the sequence of letters, and only in the older group elicited vMMN. Accordingly, automatic detection of the violation of sequential regularity indicated longer stimulus persistence in the elderly. Therefore, we can conclude that this type of inhibition also deteriorates with aging. Paradoxically, decreased inhibition led to better preattentive perception, however, this benefit diminished as attentional processes compensated the age-related differences at a higher level of processing.

## Author Contributions

All authors listed, have made substantial, direct and intellectual contribution to the work and approved it for publication.

## Conflict of Interest Statement

The authors declare that the research was conducted in the absence of any commercial or financial relationships that could be construed as a potential conflict of interest.

## Footnotes

^1^Note that in older participants in the 0 ms SOA condition we obtained long-lasting negativities. This result will be discussed later.
